# Renal Denervation as a Novel Therapeutic Option in the Acute Phase of Hemorrhagic Stroke

**DOI:** 10.1016/j.jacadv.2022.100165

**Published:** 2023-01-11

**Authors:** Francesco Versaci, Simone Calcagno, Sebastiano Sciarretta, Armando Del Prete, Massimiliano Scappaticci, Giuseppe Biondi-Zoccai

Acute hemorrhagic stroke due to intracerebral hemorrhage (ICH) represents a dramatic event with a high mortality rate. In survivors from ICH, a high percentage of severe disability has been reported. Arterial hypertension represents the most significant risk factor for hemorrhagic stroke, and an acute hypertensive response is highly prevalent among patients with ICH. According to several studies, significant blood pressure (BP) reduction seems to be safe in attenuating hematoma expansion, at least in selected patients.^1^ Rapid and safe achievement of the BP target value (≤140 mm Hg of systolic blood pressure [SBP]) in these patients is, however, challenging, and multiple-drug therapy is often not enough. The potential benefit of an antihypertensive therapy seems to be dependent on the extent, period, or method of BP lowering.

Renal denervation (RDN) is an ongoing therapeutic option for the treatment of resistant arterial hypertension,^2^^,^^3^ and preclinical studies have already shown that RDN improves outcomes in an animal model of acute ischemic stroke.^4^ In 2015, we reported originally on RDN in a compassionate critically ill patient with hemorrhagic stroke because a full antihypertensive medical therapy failed to reduce BP values, leaving a high risk of enlargement of intracranial hematoma.^5^ After completing RDN, we obtained an immediate, significant, and persistent BP reduction, as well as heart rate normalization and recovery of renal function.

Capitalizing on this first pioneering case, we performed a single-center all-comers registry including all patients admitted to our stroke unit with ICH and persistently high SBP (≥150/90 mm Hg), despite therapy with ≥4 antihypertensive drugs (AHDs) at the maximum tolerated dose. We excluded patients with arteriovenous malformation or intracranial aneurysm as the cerebral bleeding source. The RDN procedure was performed according to a standard protocol^2^ with the Symplicity Spyral multi-electrode device (Medtronic, Galway, Ireland). The primary endpoint was the effect of RDN treatment on established BP targets (<150/90 mm Hg). Details on these patients were collected as part of those included in the Global SYMPLICITY Registry DEFINE study (NCT01534299), approved by our institutional ethics committee. For analysis purposes, we report continuous variables as mean ± standard deviation and categorical variables as count (%). A 2-tailed value of *P* < 0.05 was considered statistically significant for inferential purposes.

From January 2021 to May 2022, 10 consecutive patients underwent RDN for ICH and resistant hypertension: 55% were male and most surprisingly young (age 50.8 ± 11.1 years). The mean ICH size was 63.1 ± 27.5 mm, and the most frequent location was the left intra-axial subcapsular area. Almost all patients were previously already treated for arterial hypertension with mean 2.2 ± 1.3 AHDs/patient. Angiotensin-converting enzyme inhibitors, angiotensin receptor blockers, and diuretics were the most commonly used agents. At hospital admission, the mean SBP was 197.7 ± 15.1 mm Hg, and the mean diastolic blood pressure (DBP) was 104.6 ± 7.6 mm Hg. During hospitalization, BP reduction was attempted with 5.0 ± 0.5 AHD and deep sedation, failing to achieve normal values (mean SBP 185.0 ± 11.2 mm Hg, mean DBP 94.4 ± 10.1 mm Hg), despite a significant drop in DBP in comparison to admission BP (*P* = 0.03).

**Figure 1:**
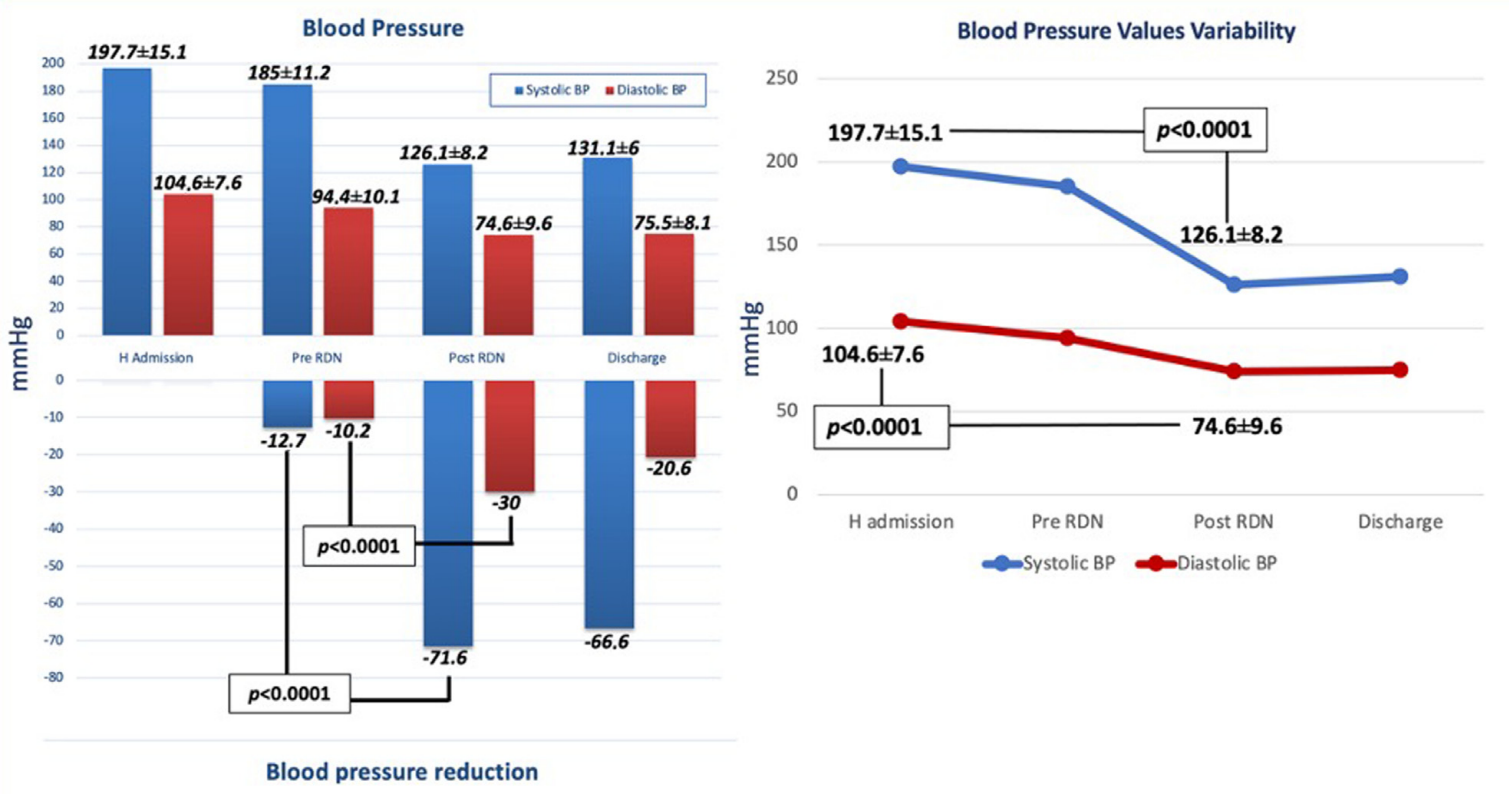
**Blood Pressure Values Trend at Different Study Times** BP = blood pressure; H = hospital admission; RDN = renal denervation.

After collegial discussion, involvement of an institutional health care committee, and obtaining written informed consent from family members, all patients thus underwent RDN within 48 hours after ICH in both main renal arteries, reaching distal branches. Ablations were completed 13.3 ± 6 times on the right renal artery and 14.2 ± 6.6 times on the left one. After the intervention, SBP decreased to 126.1 ± 8.2 mm Hg and DBP to 74.6 ± 9.6 mm Hg, with a significant drop for both (−71.6 mm Hg and −30 mm Hg, respectively, *P* < 0.001 for both) (Figure 1). During the subsequent stay, uneventful in all patients, drug therapy was reduced to 3.6 ± 0.5 AHD, without need for sedation. All subjects were safely discharged, with a mean BP of 131.1 ± 6/75.5 ± 8.1 mm Hg and 2.8 ± 1.1 AHDs as home therapy.

Arterial hypertension is the most important risk factor for ischemic and hemorrhagic stroke, yet during the acute phase of ICH, a rapid and substantial increase of BP can often be observed. The exact pathophysiologic mechanism underlying resistant hypertension in patients with ICH remains unclear. Hypothetical mechanisms include multiple and combined stress responses involving sympathetic hyperactivity, altered parasympathetic activity, increased catecholamine secretion, or a combination of these factors. Therefore, RDN in combination with maximal AHD therapy may favorably impact neuromodulation of sympathetic activity, leading to immediate BP reduction in patients with ICH. Despite our promising findings, it is important to highlight the pilot features of our present work. Indeed, as no formal comprehensive registry of patients with ICH was created at our institution, we cannot provide comparative data on individuals with ICH without hypertension, nor on those with ICH and hypertension, albeit adequately managed noninvasively.

In conclusion, this is the first clinical study on RDN in the acute phase of hemorrhagic stroke, and it suggests that RDN may lead to an immediate, significant, and persistent BP reduction and possibly improve clinical outcomes. While RDN, by improving BP control in hypertensive patients without an established cerebrovascular disease, may conceivably prevent stroke, our work suggests that RDN may also become a therapeutic option for patients with recent hemorrhagic stroke and could be considered whenever medical therapy alone is not sufficient to permanently reduce SBP values to <140 mm Hg. Irrespective of our favorable findings, additional controlled studies are required to confirm these results.
